# Evaluation of the Disintegrant Properties of Native Starches of Five New Cassava Varieties in Paracetamol Tablet Formulations

**DOI:** 10.1155/2017/2326912

**Published:** 2017-07-09

**Authors:** Frank Kumah Adjei, Yaa Asantewaa Osei, Noble Kuntworbe, Kwabena Ofori-Kwakye

**Affiliations:** Department of Pharmaceutics, Faculty of Pharmacy and Pharmaceutical Sciences, Kwame Nkrumah University of Science and Technology (KNUST), Kumasi, Ghana

## Abstract

The disintegrant potential of native starches of five new cassava (*Manihot esculenta *Crantz.) varieties developed by the Crops Research Institute of Ghana (CRIG) was studied in paracetamol tablet formulations. The yield of the starches ranged from 8.0 to 26.7%. The starches were basic (pH: 8.1–9.9), with satisfactory moisture content (≤15%), swelling capacity (≥20%), ash values (<1%), flow properties, and negligible toxic metal ion content, and compatible with the drug. The tensile strength (*T*_*s*_), crushing strength (*C*_*s*_), and friability (*F*_*t*_) of tablets containing 5–10% w/w of the cassava starches were similar (*p* > 0.05) to those containing maize starch BP. The disintegration times of the tablets decreased with increase in concentration of the cassava starches. The tablets passed the disintegration test (*D*_*T*_ ≤ 15 min) and exhibited faster disintegration times (*p* > 0.05) than those containing maize starch BP. The disintegration efficiency ratio (DER) and the disintegration parameter DER_*c*_ of the tablets showed that cassava starches V20, V40, and V50 had better disintegrant activity than maize starch BP. The tablets passed the dissolution test for immediate release tablets (≥70% release in 45 min) with dissolution rates similar to those containing maize starch BP.

## 1. Introduction 

Starch and its derivatives (native starches and modified starches, e.g., sodium starch glycolate) are principally used as disintegrants in pharmaceutical tablet formulations. Starch and its derivatives are also used as diluents, binding agents, glidants, and thickeners. Disintegrants are pharmaceutical excipients that are included in tablet formulations with the aim of facilitating the break-up of the compressed tablets into small fragments in aqueous media. The enhanced splitting of the tablets in aqueous media enhances the dissolution, absorption, and bioavailability of orally administered drugs. Other substances employed as disintegrants in pharmaceutical formulations are cellulose and its derivatives (e.g., microcrystalline cellulose, croscarmellose sodium, and low-substituted hydroxypropyl cellulose), resin and its derivatives, and crospovidone [1].

Native starches derived from botanical sources are commonly employed as disintegrants in pharmaceutical tablet formulations usually in a concentration range of 2–10% w/w [2]. The addition of starch and other disintegrants in tablet formulations may be performed intragranularly (endodisintegrants), extragranularly (exodisintegrants), or as a combination of intragranular and extragranular techniques (endo-exo-disintegrants). In intragranular addition, starch is included in the powder mixture and granulated whiles in extragranular addition dry starch powder is added external to the prepared granules. In intra- and extragranular addition, half the amount of starch is included in the tablet formulation intragranularly, and the other half extragranularly. The mode of incorporation of a disintegrant influences its disintegrant effectiveness. Starch exhibits faster disintegrant action when added extragranularly than intragranularly [2]. However, disintegrants which are added to tablet formulations both intra- and extragranularly give the best disintegrant performance [3]. Other factors which affect disintegrant effectiveness are particle size, moisture content, and the compression force applied [4].

Various mechanisms of disintegration have been proposed for disintegrants. These mechanisms which have variable application to different types of disintegrants are swelling, capillary action or wicking, strain recovery, heat of interaction, and interruption of particle-particle bonds [1, 5]. Swelling is reported as the main mechanism of disintegrant action of starch and its derivatives [1].

The first disintegrating agents to be used in tablet formulations were native starches of maize, potato, and wheat [6]. In general, native starches lack the ideal characteristics of tablet disintegrants. For instance, native starches have poor compression properties and are effective as disintegrants in rather high concentrations (10–15% w/w) compared to modified starches [1]. Genetic, physical, and chemical modification techniques have been used to change the starch granular structure of native starches to influence their properties and functionality as pharmaceutical excipients. Maize, cassava, wheat, and potato are the most important botanical sources of starch production [7] with about 10.2 million tons of cassava starch produced in 2015, mainly in Southeast Asia and Brazil [8].

The Crop Research Institute of Ghana (CRIG), Fumesua, is using genetic techniques to develop new cassava varieties with high starch, fiber, nutrient content, and other functional properties. The aim of the current study was to evaluate the disintegrant properties of native starches of five improved cassava varieties developed by CRIG, Fumesua, in paracetamol tablet formulations. Paracetamol was used as a model drug because it has poor flow and compression properties and high capping and lamination tendencies and lacks any inherent disintegrant activity. The effects of the cassava starch disintegrants on the physicomechanical and drug release properties of the formulated paracetamol tablets were evaluated.

## 2. Materials and Methods

### 2.1. Materials

Five varieties of matured fresh root tubers of cassava (*Manihot esculenta *Crantz.), namely, Sika Bankye, Ampong, AW/10/008, 12/0245, and 12/0197 (coded V10, V20, V30, V40, and V50, resp.), were obtained from CRIG, Fumesua, Ghana. The cassava varieties were planted in March 2014 and harvested in October 2015, and starch extractions were undertaken within two days of harvesting. Maize starch BP (Kathwada, Ahmedabad, India) was used as the reference disintegrant (coded V60). Paracetamol BP (Changshu Huagang Ltd., China), magnesium stearate (Anhui Sunhere Ltd., China), talc and lactose (Haicheng Pinyang Ltd., China), polyvinylpyrrolidone (PVP) (Quzhou Jianhua Nanhang Ltd., China), sulphuric acid, nitric acid, hydrochloric acid and sodium hydroxide pellets (Merck KGaA, Germany), ethanol 96% (Sasol Chemical Ltd., South Africa), and potassium dihydrogen orthophosphate (Kosdaq Co., South Korea) were used.

### 2.2. Methods

#### 2.2.1. Extraction of Starch and Determination of Moisture Content

Freshly harvested cassava tubers were washed, peeled, cut into small pieces, rewashed, and weighed. The cassava was milled into a pulpy slurry, passed through a nylon fiber, and left to stand for 12 h after which the supernatant was decanted. The cassava starch was collected, dried at 40°C for 30 min in an oven, reduced to fine powder by trituration, and passed through a 1.6 mm sieve. The percentage starch yield was calculated as follows:

(1) %  starch  yield=weight  of  dried  starchweight  of  peeled  tubers×100.

The moisture content of the dried starch was determined using the British Pharmacopoeia method [9].

#### 2.2.2. Determination of pH and Ash Values

Ten grams of cassava starch powder was weighed and added to 15 ml distilled water and mixed. Boiling distilled water was added to the mixture to make up to 100 ml. The slurry was allowed to cool and the pH was determined with a Eutech pH meter (pH 510, pH/mV/°C meter, Singapore). The total ash, acid insoluble ash, and water soluble ash of the cassava starches were determined using official methods [9].

#### 2.2.3. Assessment of Swelling Index

The tapped volume occupied by 10 g of the powdered cassava starch in a 100 ml measuring cylinder was recorded (*V*_*d*_). The starch powder was dispersed in 70 ml of distilled water and made up to 100 ml with water. After 18 hours of standing the volume of the sediment (*V*_*w*_) was determined. The swelling index (%) was calculated using the equation:

(2) $$\begin{array}{c} \text{Swelling index}=\left[\;\frac{\left(V_{w}-V_{d}\right)}{V_{d}}\;\right]\times 100. \end{array}$$

#### 2.2.4. Solubility Determination

The solubility of the cassava starch powders was determined in cold water, warm water, chloroform, and ethanol (96%) at 25°C. Five hundred milligrams of the starch powder was added to 50 ml of solvent and allowed to stand overnight. Twenty-five milliliters of the supernatant was placed in preweighed Petri dishes and evaporated to dryness over a water bath and further dried to constant weight in an oven at 100°C. The mass of the residues was determined with an analytical balance (Adam equipment, UK) and expressed as the percent solubility of the cassava starch in the respective solvents.

#### 2.2.5. Bulk and Particle Density Determination

Thirty grams (*M*_1_) of cassava starch powder was weighed into a 100 ml measuring cylinder and the volume occupied (*V*_1_) noted. The measuring cylinder was then tapped on a hard surface to consolidate the powder to a constant volume (*V*_2_). The fluff density, tapped density, Hausner ratio, and Carr's index were calculated as follows:

(3) Fluff  density=M1V1,Tapped  density=M1V2,Hausner  ratio=Tapped  densityFluff  density,Carr's  index=Tapped  density−Fluff  densityTapped  density×100.

The particle density of the starches was determined by liquid displacement method at 25°C and calculated as the weight of starch divided by the volume of the liquid it displaces [10]. Liquid paraffin was used as the displacement liquid as the starches are practically insoluble in it.

#### 2.2.6. Determination of Angle of Repose

A funnel was clamped with its tip 2 cm above a hard horizontal surface. The starch powder was allowed to flow through the funnel until the apex of the powder formed just touched the funnel's tip. The height (*h*) and the mean diameter (*D*) of the base of the starch powder cone were determined. The angle of repose (*θ*) was calculated using the equation

(4) tan θ=2hD,θ=tan−1⁡2hD.

#### 2.2.7. Determination of Toxic Metal Ion Content

The content of the toxic metal ions of cadmium (Cd), arsenic (As), lead (Pb), and mercury (Hg) in the cassava starches was determined with an atomic absorption spectrophotometer (Buck Scientific Model 210V GP) as previously reported [11, 12].

#### 2.2.8. Drug-Starch Compatibility Studies

A Fourier transform infrared spectrometer (FTIR) (PerkinElmer, UATR Spectrum 2, 941333, UK) was used to determine the compatibility between paracetamol and the cassava starches. The spectra of the individual starches, paracetamol, and the physical mixtures of the drug and starch were recorded by scanning in the wavelength region 4000–400 cm^−1^ using the FTIR spectrometer by placing the sample into a diffuse reflectance sampler. The spectra of the three samples were superimposed to assess whether or not the principal absorption bands present in the drug and starches are present in the physical mixtures.

### 2.3. Preparation of Tablets

Paracetamol granules (420 g) comprising of paracetamol (83.3%) as active pharmaceutical ingredient, lactose (0.2, 2.7, 5.2%) as diluent, polyvinylpyrrolidone, PVP (4.5%), as binder, talc (1.2%) as glidant, magnesium stearate (0.2%) as lubricant, and cassava starch or maize starch BP (5, 7.5, 10%) as disintegrant were prepared by the wet granulation method. The required amount of paracetamol was dry-mixed with lactose and starch and moistened with PVP solution and massed for 10 min in a V-blender (Cadmach Machinery Co. Pvt. Ltd., India). The damp mass was manually screened through a number 12 mesh sieve (1680 *μ*m) and dried in a hot air oven at 50°C for 2 h. The dry granules were manually screened using a number 16 mesh sieve (1190 *μ*m) and mixed with talc for 5 min in the V-blender. Finally, magnesium stearate was added and blended for a further 5 min and discharged. The different batches of the paracetamol granules in the size range of 595–1190 *μ*m were compressed into tablets (~600 mg) at pressures of 45–50 KN using a Cadmach CTX 26 tableting machine (Cadmach Machinery Co. Pvt. Ltd., India). The tablets were stored for 24 h after compression to allow for elastic recovery and hardening before evaluation.

### 2.4. Evaluation of Tablet Properties

#### 2.4.1. Uniformity of Weight Test

The uniformity of weight of twenty randomly selected tablets from each batch was determined according to the British Pharmacopoeia method [9].

#### 2.4.2. Measurement of Tablet Thickness and Diameter

The thickness and diameter of ten randomly selected tablets from each batch were determined using a digital vernier caliper (4Cr13 stainless steel digital caliper, China).

#### 2.4.3. Assay of Tablets

The paracetamol content of the tablets produced was determined using the procedure described in the British Pharmacopoeia [9]. The amount of drug in the tablets was determined spectrophotometrically (T90 UV/VIS spectrometer, PG Instruments Ltd., UK) at *λ*-max of 275 nm using the regression data of the calibration curve (*y* = 625.14*x* + 0.0259, *R*^2^ = 0.9983) of paracetamol (0.25–1.5 mg/100 ml) in 0.1 M sodium hydroxide.

#### 2.4.4. Crushing Strength and Tensile Strength Determination

The crushing strength (*C*_*s*_) of ten randomly selected tablets from each batch was determined with an Electrolab hardness tester (Maharashtra, India) as described in the British Pharmacopoeia [9]. The tensile strength (*T*_*s*_) was determined using the equation

(5) $$\begin{array}{c} T_{s}=\frac{2C_{s}}{\pi Dt}, \end{array}$$

where *D* is the diameter and *t* is the thickness of the tablet [13].

#### 2.4.5. Friability Test

The friability of the tablets was determined with an Erweka Friabilator (TA 20, GmbH, Heusenstamm, Germany). Ten tablets weighing ~6.0 g from each batch were placed in the friabilator and it was operated for 4 min at 100 rpm. The tablets were dedusted and reweighed and the difference in tablet weight was determined. The friability was calculated as follows:

(6) $$\begin{array}{c} \text{Friability}\left(\;\%\;\right)=\left[\;\frac{\left(W_{1}-W_{2}\right)}{W_{1}}\;\right]\times 100, \end{array}$$

where *W*_1_ is original weight and *W*_2_ is final weight.

#### 2.4.6. Disintegration Test

The disintegration time of the tablets of each batch was determined using an Erweka disintegration apparatus (ZT-4, Erweka, Heusenstamm, Germany) as described in the British Pharmacopoeia [9].

#### 2.4.7. Determination of Disintegration Efficiency Ratio

The disintegration efficiency ratio (DER) was determined using the relationship

(7) $$\begin{array}{c} DER=\frac{\left(C_{s}/F_{t}\right)}{D_{T}}, \end{array}$$

where *C*_*s*_, *F*_*t*_, and *D*_*T*_ are the crushing strength, friability, and disintegration time, respectively. The disintegration parameter DER_*c*_ was determined using the formula

(8) $$\begin{array}{c} {DER}_{c}=\frac{{DER}_{test}}{{DER}_{reference}}, \end{array}$$

where DER_test_ is the DER of tablets containing the test disintegrant (V10, V20, V30, V40, and V50), while DER_reference_ is the DER of tablets containing the reference disintegrant (V60). When DER_*c*_ > 1, the DER of the test cassava starch disintegrant is considered to possess better disintegrant action than the reference disintegrant and vice versa [14, 15].

#### 2.4.8. In Vitro Dissolution Tests

In vitro tablet dissolution tests were undertaken with an Erweka dissolution apparatus (Type DT6, GmbH, Heusenstamm, Germany) under sink conditions. Dissolution was determined in 900 ml phosphate buffer pH 5.8 at a paddle speed of 50 rpm and temperature of 37 ± 0.5°C. A tablet was carefully placed into each vessel to exclude air bubbles from its surface. At times 5, 10, 15, 30, 45, and 60 min, 20 ml of the dissolution medium was withdrawn and filtered and replaced with an equal volume of medium. The filtrates were diluted to 50 ml with 0.1 M NaOH and the absorbance of each solution was measured at 257 nm with a UV spectrophotometer (T90 UV/VIS spectrometer, PG Instruments Ltd., UK). The amount of paracetamol dissolved was determined using the regression data equation (*y* = 624.16*x* + 0.0283, *R*^2^ = 0.9975). Triplicate determinations were performed for each batch. Graphs of percentage drug released were plotted against time to establish the dissolution profiles of paracetamol.

### 2.5. Statistical Analysis

Differences among mean values were determined using one-way analysis of variance (ANOVA) followed by Tukey's post hoc multiple comparison test with GraphPad Prism version 5.00 for Windows (GraphPad Software, San Diego California, USA). At 95% confidence interval, *p* ≤ 0.05 was considered significantly different.

## 3. Results and Discussion

### 3.1. Extraction and Determination of Physicochemical Properties of the Cassava Starches

All the five varieties of cassava (*Manihot esculenta *Crantz.) after extraction produced starch yields ranging from 8.0% to 26.8% with the ranking of V20 > V40 > V10 > V30 > V50. Cassava starch yield was affected by the cassava variety used. Other factors which are known to affect cassava starch yield include processing factors [16], the extraction method employed [17], and the season of harvest of the cassava crop [18]. The cassava starches were white in colour and odourless and had characteristic fine texture and bland taste which complied with the official organoleptic tests for starch [9].  Table 1 presents some physicochemical properties of the cassava starches studied. The moisture content of the cassava starches was 2.1–10.0% and complied with the official specification of ≤15% [9]. The moisture content of pharmaceutical excipients affects its microbiological stability and storage, agglomeration, and flow properties [19]. The moisture content of the starches may also be influenced by their crystallinity, humidity, particle size, hygroscopic nature, and the velocity of moist air [20].

The bulk properties describe the density, consolidation, and flow of a powder mass. It also denotes how well the starch powders can be compressed since smaller particle sizes resist free flow because of adhesion between the powders [21]. The particle, fluff, and tapped densities of the cassava starches followed the same ranking of V20 > V30 > V50 > V40 > V10. It could be deduced from the results that the cassava starches studies exhibited satisfactory bulking properties for pharmaceutical use [22, 23].

The cassava starches had poor solubility in cold water, 96% ethanol, and chloroform. There was, however, marked increase in the solubility of the cassava starches in warm water. The swelling power of starches is employed to predict the swelling of tablets during disintegration test in order to release the drug for dissolution [24]. The ranking of the swelling capacity of the cassava starches was V30 > V20 > V40 > V10 > V50. The results confirm the observation that, at room temperature, cassava starches exhibit good swelling and water retention capacities and could absorb up to 30% of their weight in excess water [25]. The swelling of starch granules is attributed to the highly branched amylopectin portion and is hindered by the linear amylose portion [26], and the absence of amylose lipid complexes in cassava starches is known to enhance their swelling capacities [27, 28]. Aqueous dispersions of the cassava starches were basic in nature. The starches can preferably be employed in the formulation of alkaline drugs since there would be less tendency for drug-excipient interaction [29].

Carr's index and Hausner ratio both describe the compressibility of the starch powder while the angle of repose characterizes the flow properties of powders and is dependent on the interparticulate resistance to movement between particles [30]. Both Carr's index and Hausner ratio followed the same ranking of V20 > V50 > V30 > V40 > V10. The cassava starches generally showed good flow properties as indicated by Carr's index and Hausner ratio and the angle of repose values [22].

The toxic metal ion analysis showed the absence of mercury and an insignificant amount of the toxic heavy metals of arsenic, lead, and cadmium. This suggests the possible nontoxicity of the cassava starches and could therefore be used as pharmaceutical excipients. The total ash, water-insoluble ash, and acid insoluble ash values of the cassava starches were low which suggests that the amounts of earthly materials or adulterants present in the cassava starches are insignificant. The physicochemical properties investigated are important in determining the functional properties of the cassava starches.

**Figure 1:**
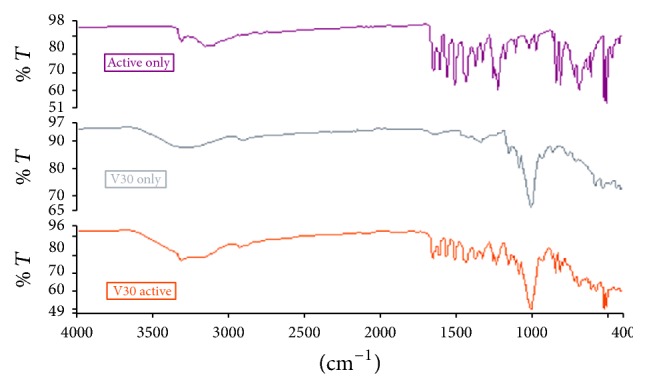
FTIR spectra of pure paracetamol (active), cassava starch (V30), and the physical mixture of paracetamol and cassava starch (V30 active).

Figure 1 presents a sample FTIR spectra of pure paracetamol powder, cassava starch (V30), and a physical mixture of the drug and the cassava starch. The spectrum of pure paracetamol structure revealed functional groups at 3322.03 cm^−1^, 3159.39 cm^−1^ (hydroxyl group, O-H stretching) and 1561.11 cm^−1^, 1504.98 cm^−1^ (amide II band) which are characteristic of the drug. The cassava starches showed intense functional groups at 3274.71 cm^−1^ (free hydroxyl group) and 1336.55 cm^−1^ and 997.37 cm^−1^ (change from crystalline to amorphous state) which characterized them as starches. The physical mixtures contained all the principal bands of the two constituents and showed no shifting of the peaks, indicating the stability and compatibility of the starches with the drug.

### 3.2. Physicomechanical Properties of the Formulated Paracetamol Tablets

Paracetamol tablets containing different concentrations of the cassava starches as disintegrants were produced by the wet granulation technique. The starch disintegrants were added to the tablet formulations intragranularly.  Table 2 presents some physical properties of the prepared paracetamol tablets. The tablets generally exhibited good physical properties with satisfactory thickness, diameter, and drug content. The tablets also passed the British Pharmacopoeia uniformity of weight test (<2 tablets ± 5% mean weight, none ± 10% mean weight, *n* = 20) [9], except formulation V50 which contained 10% w/w cassava starch. The crushing strength provides an indication of how tablets could resist breakage during storage, transportation, and handling. Crushing strength is directly correlated with disintegration and dissolution of tablets and is affected by the nature and amount of binder employed in granulation and the compression force applied during tableting. Tablets with minimal crushing strength of 39.2 N are considered satisfactory [31], and all the tablet formulations complied with this specification. The crushing strength of tablet formulations containing maize starch and cassava starches V30 and V50 showed a clear reduction with increase in starch concentration. There was, however, no clear-cut effect of disintegrant concentration on the crushing strength of tablets containing V10, V20, and V40 cassava starches. Starch disintegrants generally tend to weaken the tablet structure when used at high concentrations [32]. There was no significant differences (*p* > 0.05) between the crushing strength of tablets containing maize starch and the cassava starches.

Tablets should have strong tensile strength to withstand pressure due to handling, film-coating, and packaging but must be weak enough to allow drug release after administration. In general, the tensile strength of the tablets reduced with increase in cassava starch concentration. There were no significant differences (*p* > 0.05) between the tensile strength of tablets containing the cassava starches and that of maize starch BP, even though the tensile strength of the latter was higher. Friability test is used to evaluate the capacity of tablets to withstand abrasion and chipping associated with handling, packaging, and shipping. Apart from tablets which contained 10% w/w of cassava starch V10, all the tablets passed the friability test (tablet loss ≤ 1%). An increase in cassava starch disintegrant concentration did not produce any specific effect on tablet friability and there were no significant differences (*p* > 0.05) between the friability of tablets which contained cassava starch and those containing maize starch BP. The disintegration of a tablet is affected by the temperature of the disintegration medium, the nature and amount of binder used, force of compression, and the nature and amount of disintegrant used [22]. An increase in the concentration of cassava starch caused a reduction in the disintegration times of the tablets.. All the tablets which contained different concentrations of cassava starch passed the official disintegration test for uncoated tablets with disintegration times of ≤15 min [9]. No significant differences (*p* > 0.05) were observed between the disintegration times of tablets containing cassava starches and those containing maize starch BP.

The crushing strength-friability ratio (*C*_*t*_/*F*_*t*_) is a better measure of the mechanical strength of tablets than crushing strength as it removes the weakness related to the friability of a tablet [10]. Thus, tablets with high *C*_*t*_/*F*_*t*_ values have strong mechanical strength [33]. The disintegration efficiency ratio (DER) is also a measure of the balance between the mechanical and disintegrant properties of tablets [18]. The DER provides a better measure of tablet quality than crushing strength/friability ratio as the DER assesses the mechanical strength of a tablet by taking into consideration the negative effects on disintegration time and weakness related to friability [34]. Generally, tablets with a better balance between disintegration and binding (mechanical) properties have higher DER values [35]. From Table 3, the DER of the tablets generally increased with increase in the concentration of cassava starch. This observation is in agreement with the results of a recent study in Nigeria [18]. The rank order of the DER values of tablets containing cassava starch was V40 > V50 > V20 > V60 > V30 >V10. Thus, tablets which contained the cassava starches V40, V50, and V20 appeared to produce a better balance between their disintegration and mechanical properties than tablets produced with maize starch BP (V60), the reference disintegrant. This observation is confirmed by the DER_*c*_ values of tablets containing the cassava starches V40, V50, and V20, which were generally >1. This provides an indication that these experimental or test cassava starches had better DER values than that of the reference disintegrant. On the other hand, tablets containing cassava starches V10 and V30 generally had DERc values <1 which shows that the two cassava starches had inferior DER values than that of the reference disintegrant.

**Figure 2:**
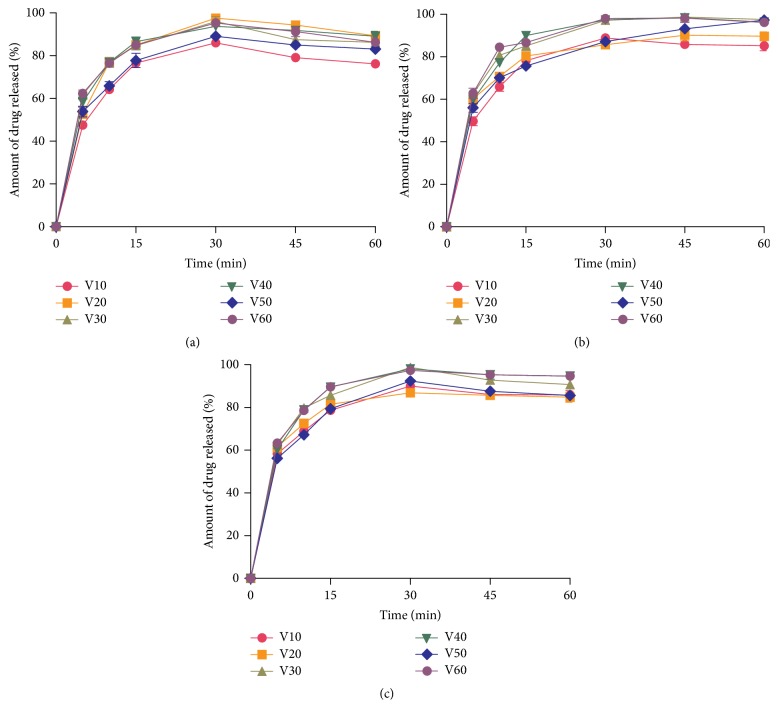
Dissolution profiles of paracetamol tablets containing (a) 5% w/w cassava starch, (b) 7.5% w/w cassava starch, and (c) 10% w/w cassava starch in phosphate buffer pH 5.8 (mean ± SD, *n* = 3).

The dissolution profiles of the paracetamol tablets containing different concentrations of the cassava starches in phosphate buffer pH 5.8 are shown in Figure 2. At 45 min, the amount of paracetamol released from the tablets containing various concentrations of cassava starch was 79–98.7%. This is in compliance with compendial requirements (drug release at 45 min ≥70%) for the dissolution of immediate release tablets [9]. Disintegration, the break-up of solid dosage forms into small, discrete particles, is an important prerequisite for the dissolution of drugs. The fast disintegration rates will accelerate the dissolution of the tablets by exposing large surface areas of the solid particles to the dissolution medium. The study has shown that starches extracted from the five new cassava varieties have similar or better disintegrant properties than maize starch BP. These cassava starches could be developed commercially as potential disintegrants and used as substitutes for the imported and more expensive maize starch BP for pharmaceutical use.

## 4. Conclusion

The results showed that the five cassava varieties have low starch yields. The starches contained negligible amounts of toxic metal ions and generally exhibited good physicochemical properties required for use as pharmaceutical excipients. The five cassava starches employed as intragranular disintegrants in concentrations of 5–10% w/w produced paracetamol tablets with satisfactory physical properties comparable with maize starch BP. There were no interactions between the starches and the active pharmaceutical ingredient and excipients used in producing the tablets. Generally, the tensile strength (*T*_*s*_) and crushing strength (*C*_*s*_) of the tablets decreased with increase in starch concentration. All tablets containing the cassava starches disintegrated in aqueous medium in less than 15 minutes and the disintegration time decreased with increase in starch concentration. The friability of the tablets was generally unaffected by an increase in starch concentration. The disintegration efficiency ratio (DER) of tablets containing the cassava starches generally increased with increase in starch concentration. Tablets containing different concentrations of the cassava starches released more than 70% of the drug in phosphate buffer pH 5.8 at 45 minutes and were comparable to tablets containing maize starch BP. Even though starch from cassava variety V40 had a rather low starch yield of 11.2%, it appeared to be the preferred cassava starch disintegrant as the different concentrations of the starch generally produced tablets with superior physicomechanical and in vitro drug dissolution characteristics.

## Figures and Tables

**Table 1:** Some physicochemical properties of starches extracted from the five varieties of cassava.

Parameter	Type of cassava starch				
	V10	V20	V30	V40	V50
Starch yield (%)	10.4	26.8	9.1	11.2	8.0
Moisture content (%)	7.20 ± 1.22^a^	2.07 ± 0.31^a^	10.00 ± 0.00	9.53 ± 0.12	9.54 ± 0.31
Ph	9.94 ± 0.01	8.07 ± 0.03	9.03 ± 0.02	9.25 ± 0.06	9.47 ± 0.09
Swelling index (%)	21.43 ± 0.00	23.72 ± 1.11	26.52 ± 1.31	21.98 ± 0.95	20.00 ± 0.00
Ash values					
Total ash (%)	0.56 ± 0.05	0.98 ± 0.07	0.68 ± 0.02	0.70 ± 0.03	0.69 ± 0.01
WSA (%)	0.048 ± 0.022	0.025 ± 0.121	0.005 ± 0.326^a^	0.037 ± 0.009	0.024 ± 0.023
AIA (%)	0.013 ± 0.007^a^	0.009 ± 0.054	0.004 ± 0.021	0.005 ± 0.033	0.003 ± 0.011
Solubility (mg/ml)					
Cold water	0.016 ± 0.000	0.060 ± 0.001^a^	0.083 ± 0.001^a^	0.015 ± 0.001	0.020 ± 0.000
Warm water	2.140 ± 0.012^a^	1.055 ± 0.001	1.460 ± 0.011^a^	1.232 ± 0.010	1.043 ± 0.002
Ethanol	0.035 ± 0.001	0.030 ± 0.001	0.068 ± 0.000	0.030 ± 0.001	0.092 ± 0.000
Chloroform	0.028 ± 0.004	0.015 ± 0.001	0.024 ± 0.001	0.052 ± 0.001	0.060 ± 0.001
Bulk properties (g/cm^3^)					
Particle density	1.833 ± 0.020	1.863 ± 0.018^a^	1.851 ± 0.000^a^	1.836 ± 0.022	1.848 ± 0.020
Fluff density	0.585 ± 0.013	0.693 ± 0.018^a^	0.625 ± 0.000^a^	0.608 ± 0.014	0.612 ± 0.000
Tapped density	0.643 ± 0.016	0.818 ± 0.025^a^	0.703 ± 0.018^a^	0.672 ± 0.017	0.682 ± 0.000
Flow properties					
Hausner ratio	1.09 ± 0.03	1.18 ± 0.04^a^	1.13 ± 0.03^a^	1.10 ± 0.05	1.14 ± 0.00^a^
Carr's index (%)	9.05 ± 2.13	15.33 ± 2.46^a^	11.10 ± 2.37^a^	9.58 ± 2.12	12.02 ± 0.00^a^
Angle of repose (°)	35.87 ± 0.76	46.57 ± 0.06^a^	41.97 ± 0.86^a^	37.87 ± 0.76	42.77 ± 0.50^a^
Particle diameter (*µ*m)	177.5 ± 0.21	175.3 ± 0.21	162.2 ± 0.10	173.7 ± 0.22	162.8 ± 0.10
Toxic metals (mg/100 g)					
Cadmium	1.650	3.300	3.750	4.200	5.100
Arsenic	0.013	0.032	0.030	0.030	0.011
Lead	0.010	0.012	0.006	0.004	0.009
^*∗*^Mercury	0.000	0.000	0.000	0.000	0.000

(a) ^*∗*^Values are below the level of detection; (b) WSA and AIA are water soluble ash and acid insoluble ash, respectively; (c) results are the mean of triplicate determinations and means in a row followed by a superscript are significantly different (*p* < 0.05).

**Table 2:** Some physical properties of paracetamol tablets produced using different concentrations of the cassava starch disintegrants.

Type of cassava starch	Starch concentration (% w/w)	Tablet weight (g) *n* = 20	Tablet thickness (mm) *n* = 10	Tablet diameter (mm) *n* = 10	Drug content (%) *n* = 4
V10	5.0	0.614 ± 0.001	4.07 ± 0.05	13.06 ± 0.00	101.72 ± 0.004
	7.5	0.574 ± 0.021	3.65 ± 0.22	13.08 ± 0.05	100.68 ± 0.003
	10.0	0.534 ± 0.060	3.90 ± 0.09	13.05 ± 0.02	98.67 ± 0.001

V20	5.0	0.614 ± 0.121	4.13 ± 0.01	13.08 ± 0.05	95.39 ± 0.004
	7.5	0.627 ± 0.090	4.21 ± 0.13	13.08 ± 0.02	102.31 ± 0.005
	10.0	0.535 ± 0.009	3.64 ± 0.07	13.10 ± 0.01	95.42 ± 0.002

V30	5.0	0.543 ± 0.122	3.76 ± 0.22	13.06 ± 0.01	103.42 ± 0.004
	7.5	0.593 ± 0.112	4.12 ± 0.10	13.03 ± 0.07	95.53 ± 0.000
	10.0	0.599 ± 0.103	4.08 ± 0.03	13.08 ± 0.03	96.87 ± 0.001

V40	5.0	0.625 ± 0.018	4.24 ± 0.07	13.09 ± 0.03	95.96 ± 0.006
	7.5	0.594 ± 0.028	3.85 ± 0.08	13.07 ± 0.01	98.86 ± 0.002
	10.0	0.583 ± 0.008	3.89 ± 0.03	13.08 ± 0.01	95.71 ± 0.004

V50	5.0	0.631 ± 0.087	4.07 ± 0.07	13.08 ± 0.01	96.35 ± 0.003
	7.5	0.600 ± 0.119	4.03 ± 0.15	13.05 ± 0.01	95.67 ± 0.004
	10.0	0.641 ± 0.011	4.21 ± 0.08	13.08 ± 0.01	100.86 ± 0.001

^*∗*^V60	5.0	0.561 ± 0.092	3.71 ± 0.12	13.07 ± 0.04	95.78 ± 0.009
	7.5	0.562 ± 0.101	3.75 ± 0.07	13.10 ± 0.05	96.65 ± 0.003
	10.0	0.541 ± 0.089	3.56 ± 0.10	13.08 ± 0.02	98.23 ± 0.003

(a) ^*∗*^V60 is maize starch BP, the reference disintegrant used.

**Table 3:** Tensile strength (*T*_*s*_), crushing strength (*C*_*s*_), friability (*F*_*t*_), disintegration time (*D*_*T*_), and DER [(*C*_*s*_/*F*_*t*_)/*D*_*T*_] and DER_*c*_ ratios for paracetamol tablets containing different concentrations of the cassava starch disintegrants.

Type of cassava starch	Starch concentration (% w/w)	*T*_*s*_ (N/cm^2^)	*C*_*s*_ (N)	*F*_*t*_ (%)	*D*_*T*_ (min)	DER	DER_*c*_
V10	5.0	75.8	63.2	0.74	12.58	6.79	0.45
	7.5	74.6	55.7	0.90	13.10	4.72	0.35
	10.0	82.6	66.0	ND	3.20	—	—

V20	5.0	91.2	77.4	0.88	14.00	6.28	0.42
	7.5	112.4	97.4	0.46	12.00	17.64	1.32
	10.0	67.7	50.7	0.43	5.00	23.58	2.49

V30	5.0	96.4	74.3	0.92	14.10	5.73	0.38
	7.5	78.0	65.8	0.89	13.06	5.66	0.42
	10.0	73.1	61.3	0.71	9.10	9.49	1.00

V40	5.0	87.2	76.1	0.28	13.40	20.28	1.35
	7.5	94.7	94.3	0.71	10.00	13.28	0.99
	10.0	69.8	55.7	0.27	4.45	46.36	4.90

V50	5.0	117.6	98.4	0.29	14.30	23.73	1.58
	7.5	84.4	69.7	0.91	11.36	6.74	0.50
	10.0	82.6	62.8	0.43	7.55	19.34	2.04

^*∗*^V60	5.0	126.0	96.1	0.45	14.20	15.04	—
	7.5	102.5	79.0	0.45	13.15	13.35	—
	10.0	87.7	64.0	0.59	11.46	9.46	—

(a) ND: not determined due to the break-up of the tablets; (b) ^*∗*^V60 is maize starch BP, the reference disintegrant used.
